# Long-term Results of Small-incision Lenticule Extraction in High Myopia

**DOI:** 10.4274/tjo.22605

**Published:** 2016-10-17

**Authors:** Yusuf Yıldırım, Cengiz Alagöz, Abdülvahit Demir, Onur Ölçücü, Mehmet Özveren, Alper Ağca, Engin Bilge Özgürhan, Ahmet Demirok

**Affiliations:** 1 Prof. Dr. N. Reşat Belger Beyoğlu Eye Training and Research Hospital, İstanbul, Turkey; 2 Kağıthane State Hospital, Ophthalmology Clinic, İstanbul, Turkey

**Keywords:** Small-incision lenticule extraction, high myopia, high-order aberrations

## Abstract

**Objectives:**

To evaluate two-year results of small-incision lenticule extraction (SMILE) for correction of high myopia.

**Materials and Methods:**

Forty-five eyes of 35 patients with mean spherical equivalent (SE) of -7.10±0.95 D who underwent routine SMILE by a single surgeon and were followed for at least 2 years were analyzed by retrospective chart review. SMILE was performed with a Visumax femtosecond laser (Carl Zeiss Meditec, Jena, Germany). Follow-up intervals were at 1, 6, 12, and 24 months after surgery. Uncorrected and best corrected distance visual acuity (CDVA), corneal wavefront measurements, and all complications were recorded.

**Results:**

After 2 years, 86% of eyes with plano target had an uncorrected distant visual acuity (VA) of 20/20 or better. Two percent of eyes lost 1 line of CDVA, while 32% gained 1 line. The mean SE after 2 years was -0.30±0.50 D. Corneal total high-order aberrations (HOA) increased from 0.43 to 0.92 μm at postoperative 12 months. There were metallic foreign bodies at the corneal interface in 1 eye of 1 patient which caused no decrease in VA.

**Conclusion:**

SMILE for high myopia seems safe and effective in light of two-year follow-up results. The procedure caused a moderate increase in HOA.

## INTRODUCTION

Current femtosecond laser technology enables the predictable, effective and safe creation of corneal lamellar incisions.^1^ Femtosecond laser systems are increasingly used in refractive lenticule extraction (RELEX). Based on how the lenticule is removed, RELEX procedures are classified as either femtosecond lenticule extraction (FLEX) or small-incision lenticule extraction (SMILE). In the SMILE procedure, an intrastromal lenticule is created with a femtosecond laser and manually removed through a small peripheral incision.^2^ SMILE is used to treat myopia and astigmatism.^3,4^

Corneal refractive surgery and phakic intraocular lens (IOL) implantation are options for the correction of high myopia.^5^ The use of SMILE for high myopia presents several advantages, namely avoiding the flap created during laser-assisted in situ keratomileusis (LASIK) and subsequent risk of ectasia, the risk of haze in high-myopic patients after photorefractive keratectomy and the invasive procedure on phakic IOL surgery.

In this study we aimed to evaluate the long-term visual and refractive outcomes, the effect on high-order aberrations (HOA) and the complications of SMILE for the correction of high myopia.

## MATERIALS AND METHODS

This retrospective study included 45 eyes of 35 patients who underwent SMILE in the Refractive Surgery unit of the Beyoğlu Eye Research and Training Hospital between 2011 and 2013. All procedures were performed by the same surgeon. The study was performed in accordance with the Declaration of Helsinki and was approved by the Beyoğlu Eye Research and Training Hospital Ethics Committee. Informed consent forms were obtained from all patients.

All patients included in the study were over 18 years old, had a spherical equivalent (SE) refraction value over 6.0 diopters (D), and had myopia or myopic astigmatism. Other inclusion criteria were absence of other ocular disease, having normal topography and regular retinoscopic reflex, a smallest pachymetry value greater than 500 μm, having stable refraction for the previous 2 years, and a cylindrical value smaller than 1.5 D. Prior to the procedure, the regularity of each patients’ topography pattern was confirmed using a Sirius™ topography system (Costruzione Strumenti Oftalmici, Firenze, Italy). Mesopic (4 lux) pupil diameter was 6.5 mm or less in all patients. The calculated residual stromal bed thickness was greater than 250 μm.

### Pre- and Postoperative Evaluations

Visual acuity (VA) was assessed using illuminated Early Treatment Diabetic Retinopathy Study chart (Optec 3500 Vision Tester, Stereo Optical Co., USA). Objective cycloplegic refraction measurements were done using an autorefractometer (KR-1 Auto Kerato-Refractometer, Topcon, Japan). The Sirius^®^ corneal topography and aberrometry system (6 mm pupil diameter, Costruzioni Strumenti Oftalmici, Italy) was used for corneal topography, dynamic infrared pupillography, ocular wavefront analysis and corneal wavefront analysis.

IOP was measured with a Goldmann applanation tonometer. All patients underwent detailed slit-lamp examination of the anterior and posterior segments.

### Surgical Technique

All procedures within the scope of this study were performed by the same surgeon using the VisuMax^®^ (Carl Zeiss Meditec, Germany) femtosecond laser platform using the same laser settings. Spot size was 3 μm for lamellar incisions and 2 μm for sidecuts. Other settings were as follows: spot energy was 140 nJ, minimum lenticule edge thickness was 15 μm, lenticule sidecut angle was 120˚ and optical zone was 6.5 mm. The cap was planned to have a diameter of 7.5 mm with a 50˚ side cut in the superior region. A small interface was used for all patients. After making the lenticule cut and sidecut and moving the patient under the surgical microscope, a blunt spatula was used to enter the area of anterior lamellar photodisruption and remove any residual material. The same procedure was performed on the posterior lamellar photodisruption surface. After ensuring the complete separation of the lenticule from the overlying and underlying stroma, the lenticule was removed through the sidecut using forceps.

### Statistical Analysis

Mean and standard deviation were used in descriptive statistical analyses. Normality of data distribution was determined by Kolmogorov-Smirnov test. Dependent samples t-test was used to analyze repeated measures. Statistical Package for the Social Sciences (SPSS) version 20.0 (IBM Corporation, USA) software was used for all analyses.

## RESULTS

### Visual and Refractive Results

Uncorrected VA was 1.45±0.17 logMAR before the procedure, compared to 0.03±0.04 at 12 months after the procedure and 0.03±0.07 logMAR at 24 months after the procedure (p<0.001 for both). Corrected VA (CVA) was 0.06±0.08 logMAR before the procedure, compared to 0.01±0.02 at 12 months after the procedure and 0.01±0.03 logMAR at 24 months after the procedure (p<0.001 for both). Pre- and post-procedure VA values are summarized in Table 1.

**Figure 1:**
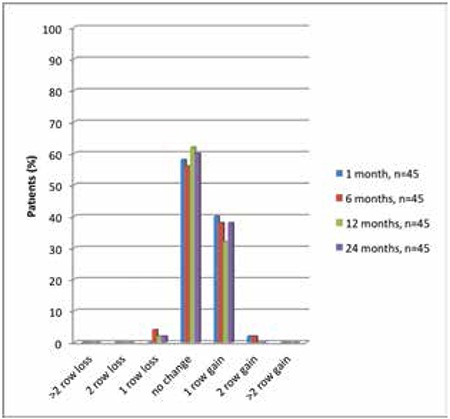
Rates of visual acuity gains and losses (in rows) in postoperative follow-up

As illustrated in Figure 1, at 1 year after the procedure, CVA decreased by 1 row in 2% of eyes and increased by 1 row in 32%. At 24 months after the procedure, CVA decreased by 1 row in 2% of eyes and increased by 1 row in 38%.

**Figure 2:**
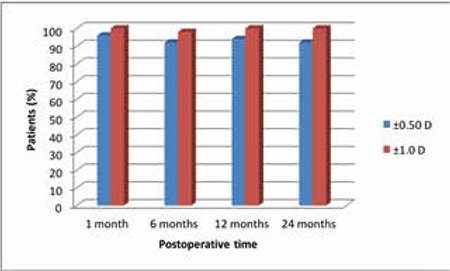
Refractive deviation from target spherical equivalent in postoperative follow-up

Emmetropia was the goal in all 45 eyes. At 1, 6, 12 and 24 months, VA was 20/20 or better in 78%, 82%, 88% and 86% of eyes, respectively. At final examination, VA was 20/25 or better in 96% of the eyes (Table 2). At 12 months after the procedure, 94% of the eyes were within ±0.5 D of the objective refraction and 100% of patients were within ±1.0 D. At 24 months after the procedure, 92% of the eyes were within ±0.5 D of the objective refraction and 100% of patients were still within ±1.0 D (Figure 2).

There were significant differences between baseline and postoperative 24-month values for SE, spherical value and cylindrical value (p<0.001 for all). Pre- and postoperative refractive values are presented in Table 3.

### Corneal High-order Aberrations

Total corneal HOA increased from 0.43±0.10 μm before the procedure to 0.92±0.17 μm at 12 months after the procedure. Mean spherical aberration was -0.20±0.05 μm preoperatively and -0.56±0.2 μm at 12 months postoperatively (p<0.001). Mean coma aberration was 0.25±0.01 μm preoperatively and 0.66±0.3 μm at 12 months postoperatively (p<0.001), while trefoil aberration was 0.20±0.1 μm preoperatively and 0.22±0.1 μm at 12 months postoperatively (p<0.04) (Table 4).

### Side Effects and Complications

**Figure 3:**
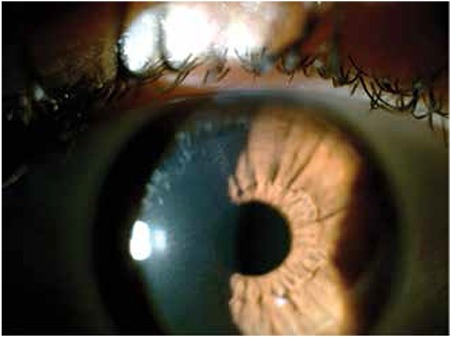
Postoperative 1-month photograph of eye that had intraoperative tearing of the side cut; metallic particles beneath the side cut and healed tear are visible

Suction loss occured in 1 eye of 1 patient while performing the cap cut. The procedure was completed successfully after suction was restored. In 1 eye of 1 patient, a 1 mm tear occured in the sidecut intraoperatively, while removing the lenticule. In the same eye, metallic deposits were observed postoperatively in the interface near the sidecut (Figure 3). None of the patients exhibited corneal epithelium ingrowth or topographic signs of corneal ectasia during follow-up.

## DISCUSSION

SMILE is a new femtosecond laser-based keratorefractive surgical procedure used to treat myopia without the creation of a flap, unlike the LASIK and FLEX procedures.^6^ There are few studies on the long-term effects of SMILE for the correction of high myopia.^7^ In the present study we evaluated the results from 2 years of follow-up from high myopic patients who underwent SMILE.

There are many studies in the literature reporting short-term outcomes of SMILE for myopia correction and comparing SMILE with FLEX and LASIK.^8,9,10^ Vestergaard et al.^10^ investigated the short-term results of 35 patients who underwent FLEX in 1 eye and SMILE in the fellow eye. The patients’ preoperative mean SE value was -7.6±1.0 D; VA was 20/40 or better in 90% of patients at postoperative day 1, and 100% at 6 months. At postoperative month 6, there was a significant improvement in CVA of about 1.5 rows. None of the eyes had more than 2 rows of gain or loss. The SMILE group achieved a postoperative mean refractive value of -0.09±0.39 D. After both procedures, final refraction at postoperative month 6 was within ±0.50 D in 88% of eyes. In another study, Vestergaard et al.^11^ performed SMILE in a randomly selected eye of 144 patients and followed them for 3 months. Forty percent of patients had a VA better than 0.1 logMAR at postoperative day 1, compared to 73% of patients at 3 months. CVA ranged from -0.01 logMAR to -0.03 logMAR. One patient gained 2 rows, 24 patients gained 1 row, and 6 patients lost 1 row of VA. The patients’ mean SE was -7.18±1.57 D preoperatively and reached -0.09±0.5 D by final follow-up examination. Final refraction values were within ±0.50 D in 77% of patients and within ±1.00 D in 95%. Ivarsen et al.^6^ evaluated the 3-month CVA results in 1,547 patients who had SMILE in both eyes. The patients’ mean SE was -7.25±1.84 D preoperatively and their postoperative refraction was -0.09±0.5 D. After 3 months, CVA was better or the same in 86% of patients. A loss of more than 2 rows was observed in 1.5% of the patients. The refractive and visual outcomes found in our study are consistent with those reported in these short-term studies.

### Study Limitations

A recent study by Pedersen et al.^7^ evaluating the 3-year results of SMILE in high-myopic patients revealed a mean SE of -7.30±1.40 D preoperatively, -0.30±0.50 D at postoperative 3 months and 0.40±0.60 D at postoperative 36 months. At the end of the 3-year follow-up period, 78% of their patients had an SE within ±0.50 D and 90% within ±1.00 D. This large study demonstrated that postoperative CVA continued to improve for 3 years after the procedure. Similarly, we observed that CVA was significantly higher in postoperative follow-up examinations compared to preoperative values. Pedersen et al.^7^ proposed restructuring of the corneal stroma, neural adaptation or the reduction of corneal haze over time as possible explanations for this phenomenon. The small number of cases in our study may also have negatively impacted our statistical evaluation.

Increases in corneal HOA adversely influence visual outcomes due to glare, halo and reduced contrast sensitivity.^7^ Corneal refractive surgery is known to increase corneal HOA.^12^ Many studies have analyzed changes in corneal HOA following SMILE.^13,14^ Sekundo et al.^13^ performed SMILE in 10 myopic patients and evaluated corneal HOA occurring in the 5 mm pupillary zone over a 6-month follow-up period. Total HOA were 0.18 μm preoperatively and 0.21 μm postoperatively, which was not a statistically significant change.^13^ Shah et al.^14^ performed SMILE in 51 eyes of 41 patients and evaluated changes in ocular wavefront after 6 months. They found that total HOA increased significantly from 0.19 μm preoperatively to 0.32 μm at 6 months postoperatively (p=0.01). They also observed significant increases in coma (0.13 to 0.20 μm) and spherical aberrations (0.06 to 0.17 μm). In Pedersen et al.’s^7^ evaluation of the 5 mm zone of high-myopic patients, they found a significant increase in corneal HOA postoperatively but showed that the amount of aberration decreased over the long term. They attributed this to corneal restructuring following SMILE. In the present study, we observed a significant increase in corneal HOA at postoperative 12 months. We believe the higher rate of HOA in our study compared to other studies may be related to our use of a 6 mm pupillary diameter.

Agca et al.^8^ compared total corneal HOA between eyes in 20 patients who underwent SMILE in one eye and LASIK in the fellow eye. They found that total HOA, coma, spheric aberrations and trefoil aberrations were significantly increased in both groups at the end of follow-up. We also observed significantly higher total HOA, coma, trefoil and spheric aberrations at postoperative 12 months.

This increase in HOA may be a result of the composition of our study group, which included patients with high myopia, or the fact that treatment did not involve wavefront-based correction.

Many intra- and postoperative complications have been reported for the SMILE procedure.^6^ These include abrasion at the incision site, tears, difficulty extracting the lenticule, cap perforation and foreign bodies in the interface. No sight-threatening complications occured in our study. In 1 eye of 1 patient, a 1 mm tear occured in the sidecut while removing the lenticule and metallic particles from the surgical spatula were later detected in the interface near the sidecut. None of the patients exhibited corneal ectasia in the 2-year follow-up period.

Some limitations of this study were the retrospective collection of data by chart review, a small patient population, not comparing the results of high-myopic patients to those with low or moderate myopia, and not comparing SMILE with other procedures (LASIK, phakic IOL, etc.) that can be applied in high myopia.

## CONCLUSION

In this study we have demonstrated that correcting high myopia with SMILE is safe and effective in the long-term, but the procedure significantly increases corneal HOA.

### Ethics

Ethics Committee Approval: The study were approved by the Beyoğlu Eye Training and Research Hospital Local Ethics Committee (retrospective study, protocol number: 15), Informed Consent: Consent form was filled out by all participants.

Peer-review: Externally and internally peer-reviewed.

## Figures and Tables

**Table 1:**
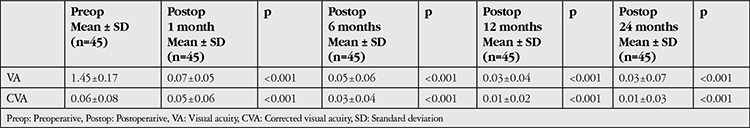
Pre- and postoperative visual acuity

**Table 2:**
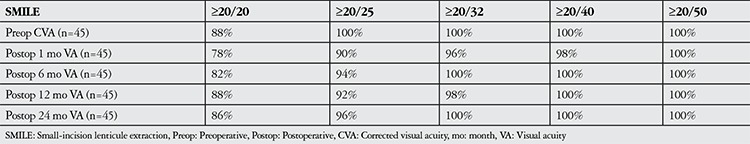
Relative comparison of postoperative visual acuities

**Table 3:**
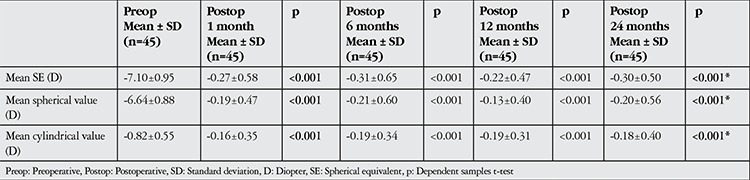
Pre- and postoperative refractive values

**Table 4:**
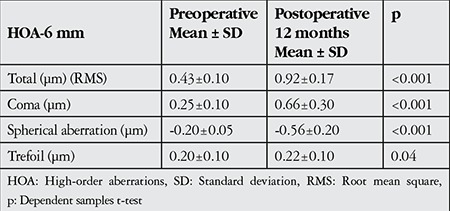
Pre- and postoperative high-order corneal aberrations
